# A survey to document toxic hazards in the zone surrounding volcanoes national park, a habitat for mountain gorillas, an endangered wildlife species in Rwanda

**DOI:** 10.3389/fvets.2023.1320162

**Published:** 2024-01-03

**Authors:** Enock Kwizera, Wilson K. Rumbeiha, Kizito Nishimwe, Julius Nziza

**Affiliations:** ^1^Department of Veterinary Medicine, University of Rwanda, Nyagatare, Rwanda; ^2^Department of Molecular Biosciences, University of California, Davis, Davis, CA, United States; ^3^Department of Food Science and Technology, University of Rwanda, Musanze, Rwanda; ^4^Gorilla Doctors, Musanze, Rwanda

**Keywords:** chemicals, wildlife, mountain gorillas, VNP, pesticides, pharmaceuticals, poisonous plants, toxicants

## Abstract

**Introduction:**

In recent years, Volcanoes National Park has seen a rise in its wildlife population, primarily due to the diligent efforts of the Rwandan government in safeguarding endangered species, notably the mountain gorillas (*Gorilla beringei spp. beringei*). This population growth has led to a pressing need for more expansive habitats, ensuring these creatures have ample space, sustenance, and shelter for their wellbeing. Consequently, there are planned park expansion activities on the horizon. However, before initiating this expansion, a critical prelude involves identifying potential threats, particularly toxic substances stemming from agricultural activities in the surrounding environment of Volcanoes National Park.

**Methods:**

To address this concern, a comprehensive study was conducted, aimed at pinpointing potential toxic hazards and assessing the awareness of the local population regarding the harm these hazards pose to wildlife species. Data was collected from individuals with no prior knowledge of the study using a pre-tested questionnaire. The questionnaire was divided into three sections: socio-demographic issues, potential toxic hazards assessment, and a section to determine awareness and risk of potential toxic hazards to humans, animals, and the environment. Respondents were selected based on specific criteria, which included being 18 years or older and residing within the National Volcano Park (NVP) area.

**Results:**

The study's findings revealed four main categories of potential toxic hazards, which include household chemicals, pharmaceutical products, agricultural pesticides, and poisonous plants. These hazards could jeopardize the health and survival of wildlife species if they consume or come into contact with them. Furthermore, the study exposed an inadequacy in the knowledge and skills of the local community in preventing these toxic hazards, which can result in death of wildlife species and ecosystem contamination and degradation.

**Conclusion:**

Study results also underscored the significance of education and training in enhancing the awareness of local communities concerning these toxic threats. Therefore, it is imperative to implement immediate measures to mitigate the adverse effects of these toxic hazards on wildlife species, especially in light of the planned park expansion.

## 1 Introduction

The Volcanoes National Park (VNP) is situated in Northern Rwanda and is part of the Virunga Massif landscape that also consists of the Mgahinga Gorilla National Park in Southwestern Uganda and the Virunga National Park in the Eastern Democratic Republic of Congo (DRC). The VNP, with an area of 160 km^2^, encompasses high-altitude volcanic mountains (ranging from 2, 400 to 4, 500 m above sea level), namely Sabyinyo, Gahinga, Muhabura, Bisoke and Karisimbi, with the highest peak at 4, 507 m above sea level (1). As a part of the Albertine rift (The western branch of the East African Rift, covering parts of Uganda, DRC, Rwanda, Burundi and Tanzania, extending from the northern end of Lake Albert to the southern end of Lake Tanganyika), this unique habitat is one of the most biologically diverse regions in the world, supporting various wildlife species, including the endangered mountain gorillas according to the International Union for Conservation of Nature (IUCN) (2, 3). The VNP is a legally protected area by the Government of Rwanda (GoR) to shield endangered species including mountain gorillas (4). In addition, eco-tourism to the VNP generates foreign income, primarily from mountain gorilla-based tourism (5, 6). Thus, the VNP is a world treasure.

Over the last few years, the wildlife population in VNP has increased. For instance, it was estimated that the wildlife population of the Virunga Mass if would increase by around 3% annually, including the 4.4% growth rate of the mountain gorillas under observation (7), thanks to the GoR's efforts to protect those endangered species. As the population has increased, mountain gorillas tend to move out of VNP to the surrounding agricultural communities, which unfortunately creates conflicts with the local communities. To better protect these endangered species, the GoR has initiated the expansion of VNP to the surrounding agricultural communities (8). These communities traditionally use agricultural and household chemicals, pharmaceuticals, and other hazardous chemicals for different reasons. The surrounding areas also contain natural toxins such as poisonous plants and mushrooms. Like communities anywhere in the world, people living in the area surrounding the VNP plant ornamental and/or herbal plants, some of which have toxic properties (9). Therefore, there are concerns that the expansion of VNP to the surrounding agricultural territory will expose the wildlife in the protected area to toxic hazards that will harm or kill them. Some published studies show that farmers in Musanze District, where VNP is located, heavily rely on pesticides to protect their crops from pests and diseases, with some using up to 12 different types of pesticides (10–12). Such heavy use of pesticides in agriculture, particularly in Musanze District, Rwanda, has been a cause for concern because of the potential of these chemicals to harm human health and the environment if they are not well managed or disposed of.

Therefore, the goal of this study was to identify the toxic hazards to wildlife in the future expansion zone surrounding VNP, and to assess people's awareness of the harm these toxic hazards pose to wildlife. We tested the hypothesis that people living in the area surrounding the VNP were not aware of the potential for these toxicants to harm or kill wildlife. This is important because the VNP harbors endangered wildlife species, such as mountain gorillas which must be conserved. Information obtained from this study is crucial for safe expansion of VNP into contiguous areas. Expanding wildlife habitat will provide wildlife species in VNP with sufficient sources of food and shelter, reduce competition, and allow wildlife will thrive in a conductive safe environment.

## 2 Materials and methods

### 2.1 Description of the study area

**Figure 1:**
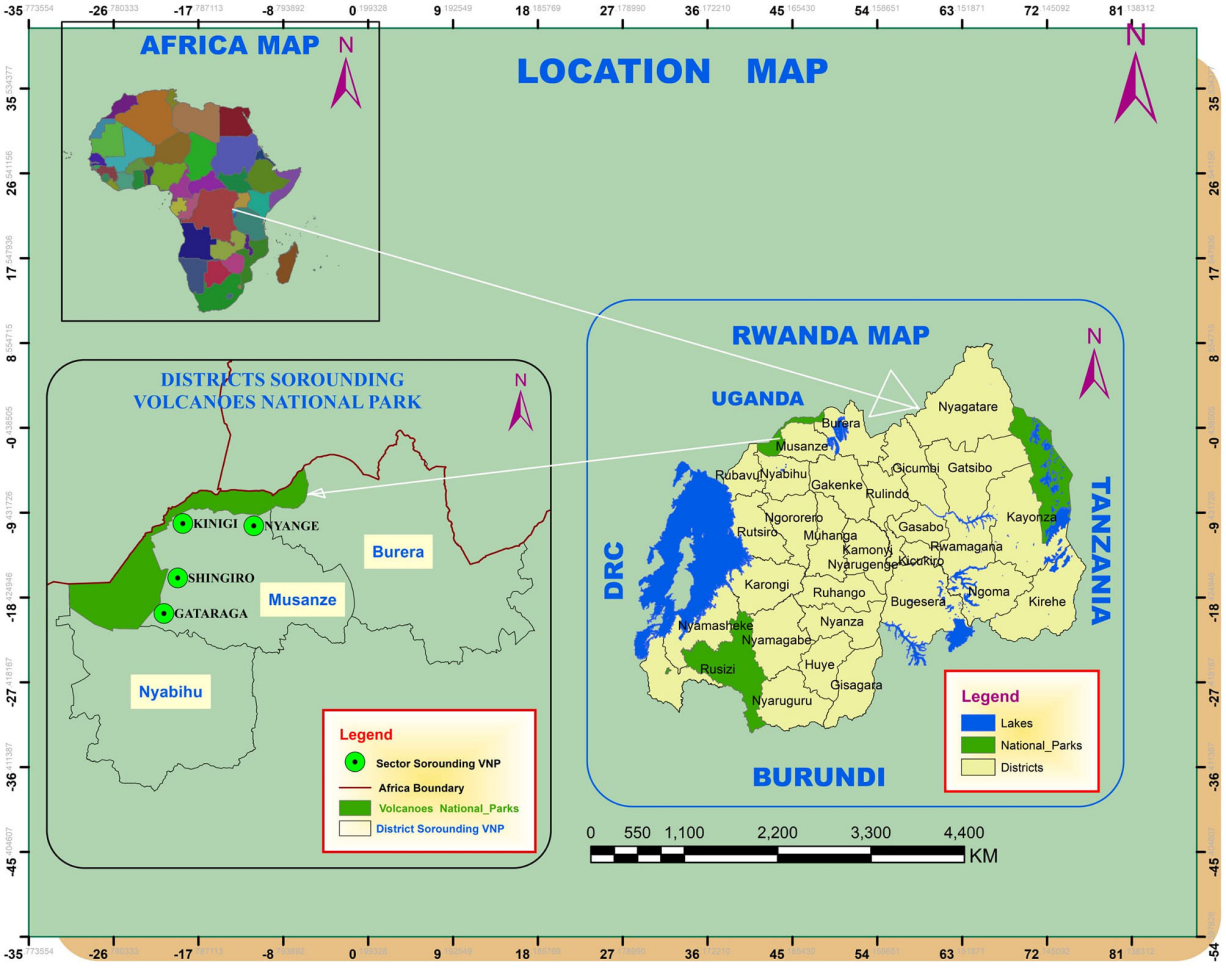
Map showing the administrative boundaries surrounding Volcanoes National Park.

The study area is located in the northern and western provinces of Rwanda. This area is near the borders with the DRC and Uganda. The study was conducted in four sectors surrounding VNP in Musanze District, namely Nyange, Kinigi, Shingiro, and Gataraga (Figure 1). The primary economic activity in the area is agriculture, with Irish potatoes, pyrethrum, wheat, onions, nuts, and maize as major cash crops cultivated (13, 14). Livestock production is mainly cattle (*Bos taurus*), sheep (*Ovis aries*), and goats (*Capra aegagrus hircus*). Beekeeping is also practiced on a small scale. The study area is characterized by heavy pesticide use to boost crop agricultural production (15, 16).

### 2.2 Sample size determination

Determination of sample size of study participants for this study was based on the estimated population of 63, 972 individuals living in the mentioned sectors surrounding VNP (16). A confidence level of 95% and a margin of error of 5% were used to determine the sample size. The Survey Monkey formula was employed, with a z-score of 1.96 (17). The calculated minimum sample size was determined to be 380 participants, representative of the entire population.

### 2.3 Target population

The target population was individuals living in the area surrounding VNP, regardless of their occupation. The only inclusion criteria were that the participants were at least 18 years old. Therefore, the study aimed at capturing a diverse range of individuals living in the vicinity of the park, including farmers and non-farmers.

### 2.4 Study design and sampling method

A cross-sectional study was conducted for identification of potential toxic hazards through interviews with people who lived in the area surrounding VNP. Non-probability (empirical) sampling using the quota approach was used because all study subjects in the research population were not available at the same time. By skipping 3rd or 4th home, survey was conducted until the required number of study subjects was reached.

### 2.5 Data collection

Data collection was conducted using a questionnaire from November to mid December 2022. Respondents were not pre-informed to avoid biased responses, and the questionnaire was designed based on previously published studies (18–21). Prior to the study, the questionnaire was piloted on a small sample of farmers (22), and questions' clarity and appropriateness were evaluated and edited accordingly. The questionnaire was divided into three main sections: socio-demographic issues, potential toxic hazards assessment, and a section to assess awareness and risk of potential toxic hazards to humans, animals, and the environment. The target respondents were selected based on specific criteria namely being 18 years old or above and living in the area surrounding VNP. The questionnaire consisted of closed questions, and 14 questions were selected to assess the respondents' level of knowledge about potential toxic hazards to wildlife species in the area surrounding VNP (Supplementary material). The questionnaire was inserted into the Kobo Toolbox for quick and reliable data collection (23). The interviews were conducted in the local language (Kinyarwanda), and verbal consent was obtained from all participants involved in the study to keep their anonymity. This study was approved by the University of Rwanda (The office of the director of research & innovation) and Rwanda Development Board (RDB) in charge of wildlife protection. Validation of names of pesticides and pharmaceutical drugs used by the participants in each study area was done by contacting local pesticide retailers and local health centers and pharmacies. The printed photos of listed poisonous plants were shown to respondents during interview in order give accurate data. Furthermore, an android application called LEAF SNAP was used to confirm the identity of plant species by scanning plants' photos (23).

### 2.6 Data analysis

The raw data collected were first exported into Microsoft Excel (version 2021) and reviewed for the accuracy. The data were then coded, entered, and verified to minimize the risk of errors. The statistical software package, SPSS v.20 (Chicago, IL), was used for data analysis in accordance with the study's objectives. The precision level was set at 5% and the confidence interval at 95%. Descriptive statistics were used to calculate the frequencies and percentages of the responses. Logistic regression was conducted to determine the association between the dependent variable, knowledge, and the independent variables, education level, and training status. To evaluate the level of knowledge of the respondents regarding potential toxic hazards around VNP, the 14 questions were scored, with a score of 1 indicating sufficient knowledge and a score of 0 indicating insufficient knowledge. The total number of points was 14. Respondents who scored >10 points were considered to have sufficient knowledge. Those who scored 7 but <10 points were considered to have moderate knowledge. Those who scored < 7 points were regarded as having insufficient knowledge about potential toxic hazards.

## 3 Results

### 3.1 Social demographic characteristics of respondents

The results of this study showed that the majority of the respondents were farmers (99.9%) with a large proportion practicing both crop and livestock farming (43.9%). The respondents were predominantly married (87.8%) and had different family sizes, with almost half of them belonging to households of 1–5 people (49.9%). In terms of educational background, the majority attended primary school (51.9%) and a considerable number had no formal school education at all (22.3%). The age bracket of the majority of respondents was youths, 31–35 years (19.1%). The study had almost an even representation of both genders, with females constituting slightly over half of the respondents (50.1%). The study was conducted in four sectors of the Musanze district, with Kinigi sector having the highest number of respondents (34%) (Table 1).

**Table 1:** Social demographic characteristics of respondents.

**Respondent's characteristics**		**Frequency (n)**	**Percentage (%)**
**Location**			
	Kinigi	139	34.4%
	Nyange	72	17.9%
	Shingiro	126	31.3%
	Gataraga	66	16.4%
**Gender**			
	Female	202	50.1%
	Male	201	49.9%
**Age**			
	18–25	55	13.6%
	26–30	60	14.9%
	31–35	77	19.1%
	36–40	64	15.9%
	41–45	37	9.18%
	46–50	28	6.95%
	51–55	21	5.21%
	56–60	25	6.21%
	61–65	13	3.24%
	66–70	9	2.23%
	71–75	5	1.25%
	76–80	9	2.23%
**Occupation**			
	Farmer	400	99.25%
	Pastor	1	0.25%
	Technician	1	0.25%
	Constructor	1	0.25%
**Marital Status**			
	Divorced	6	1.49%
	Married	354	87.84%
	Single	18	4.47%
	Widower	25	6.2%
**Family Size**			
	1–5 People	201	49.9%
	6–10 People	75	31.5%
	11–15 People	127	18.6%
**Educational Background**			
	No formal education	90	22.3%
	Primary	209	51.9%
	Secondary	100	24.8%
	College and University	4	1%

### 3.2 Household chemicals

The survey results revealed that households in the vicinity of the VNP had a low usage level of house hold chemicals. Bathroom, toilet cleaners, disinfectants, and surface cleaners were used by only 0.20% of respondents. Deodorants and sprays, bleach, perfumes, nail polish, nail polish remover, hair dyeing and hair styling products, and hair removers were not used at all. In the kitchen, dishwashing detergents and powders were used by 49.0% of respondents. Chemicals used in laundry, such as washing detergents were used by respondents at a high level of 98% (Table 2). Sleeping aids, painkillers, and cough and cold medicines were used only by 29% of respondents, while cosmetics (lotions) were used at a level of 70.5% (Table 3).

**Table 2:** Household chemicals used for laundry in communities around VNP.

**Itemized household chemicals**	**Frequency (n)**		**Percentage (%)**	
	**Yes**	**No**	**Yes**	**No**
Washing detergents	395	8	98%	2%
Bleach	0	403	0%	100%
Solvents	0	403	0%	100%
Pet flea powders and shampoo	0	403	0%	100%
Metal and wood polish	0	403	0%	100%
Washing soap	1	402	0.20%	99.80%
Antiseptics	1	402	0.20%	99.80%

**Table 3:** Household chemicals used in bedrooms in communities around VNP.

**Household chemicals**	**Frequency (n)**		**Percentage (%)**	
	**Yes**	**No**	**Yes**	**No**
Usage of chemicals in your bedrooms	286	116	71.1%	28.9%
Sleeping tablets painkillers and cough and cold medicines	2	401	0.0.5%	99.5%
Cosmetics lotions	284	119	70.5%	29.5%
Insect repellents	0	403	0%	100%

### 3.3 Pharmaceutical drugs used in humans

The findings from the survey revealed the use of various human pharmaceutical drugs. The most commonly used pharmaceutical drugs were antimalarial drugs such as Coartem^®^ (artemether/lumefantrine) which was used by 8.4% of survey participants. The usage of anthelminthic and antiprotozoal drugs was also common. For example, Metronidazole was used by 96.5% of participants, followed by Tinidazole at 96.8% and Albendazole at 97.3%. Antibiotics such as Amoxicillin were also commonly used, with a usage level of 99%, followed by Erythromycin at 26.6% and Cotrimoxazole at 13.3%. Non-steroidal Anti-inflammatory drugs such as Paracetamol and Ibuprofen were used at a very high level of 99.5% and 98.5% of survey participants respectively (Table 4). Results showed that survey participants use pharmaceutical drugs at different frequencies. Those who use pharmaceutical drugs more than once per week were at 4.5%, while those who use pharmaceutical drugs once per week were at 1.2%. The majority of the population surveyed use drugs less frequently, with 40.4% using them once every 3 months, 16.1% using them once every 6 months, and 31.3% using them once a month. The lowest percentage of pharmaceutical drug users was those who use them once per year, at 6.5%.

**Table 4:** Itemized household human pharmaceuticals used around VNP.

**Medicines category**	**Medicine**	**Frequency (n)**		**Percentage (%)**	
		**Yes**	**No**	**Yes**	**No**
**Antimalarial drugs**					
	Coartem^®^	34	369	8.4%	91.6%
**Anthelmintic/ antiprotozoal drugs**					
	Metronidazole	389	14	96.5%	3.5%
	Tinidazole	390	13	96.8%	3.2%
	Mebendazole	34	369	8.4%	91.6%
	Albendazole	392	11	97.3%	2.7%
	Nystatin	4	399	1%	99%
**Antibiotics**					
	Amoxicillin	399	4	99%	1%
	Erythromycin	107	296	26.6%	73.4
	Cotrimoxazole	53	350	13.2%	86.8%
**NSAID for fever and headache**					
	Paracetamol	401	2	99.5%	0.5%
	Ibuprofen	397	6	98.5	1.50%

### 3.4 Disposal of household chemicals and pharmaceutical drugs

The results showed that people have adopted different methods for disposal of unused household chemicals, pharmaceutical products, and residues. The majority of the respondents (40.1%) reported that they simply throw away unused chemical and packaging materials into dumpsites, while a very small proportion (0.2%) reported wrapping the materials in separate containers before discarding in the open space. Additionally, 15.6% of respondents reported burying the materials, while the majority of respondents (60.8%) reported outdoor burning the materials.

### 3.5 Use of veterinary drugs around VNP

The use of Albendazole and Ivermectin at 100% indicates that parasitic infections are the most common diseases affecting livestock in the area. Oxytetracycline, used to treat bacterial infections is an important veterinary drug used by 38% of respondents. Multivitamin supplements used to improve the general health of the livestock was used by all survey participants (100%). Vaccines were used at 55.8% to prevent certain diseases such as Rift Valley Fever, Lumpy Skin diseases, Anthrax, and so on (Table 5). The low usage of disinfectants at 17% suggests a lack of awareness of the importance of hygiene in preventing spread of diseases. The use of Limoxin^®^ 25 (Oxytetracycline 2.5% spray). Spray at 39.2% and Eye Ointments at 4.5% indicates that eye infections are also prevalent in the area. Streptomycin at 70.2% is used to treat bacterial infections, but its frequent use can lead to the development of antibiotic resistance, which is a major concern for public health.

**Table 5:** Veterinary pharmaceuticals commonly used by farmers around VNP.

**Veterinary drugs**	**Frequency (** ***n*** **)**		**Percentage (%)**	
	**Yes**	**No**	**Yes**	**No**
Albendazole	403	0	100%	0%
Oxytetracycline	153	250	38%	62%
Ivermectin	403	0	100%	0%
Multivitamin	403	0	100%	0%
Vaccines	225	178	55.8%	44.2%
Disinfectant	70	333	17%	82.6%
Limoxin^®^ 25 Spray	158	245	39.2%	60.8%
Eye Ointment	18	385	4.50%	95.5%
Streptomycin	141	262	35%	65%
Streptomycin Injection	283	120	70.2%	29.8%

### 3.6 The fate of veterinary drug residues and their packaging materials

We also studied the fate of unused veterinary drug and their packaging materials to understand the level of environmental contamination from this class of chemicals. The results showed that 46.4% of respondents discard unused veterinary drugs and their packaging materials in the open at the edge of farms 13.2% bury them, 5.5% discard them in public landfills, and 1.7% dispose of them in streams. Interestingly, a significant proportion (62.8%) reported outdoor burning the unused veterinary drugs and packaging materials, which could potentially release harmful chemicals into the environment. These findings suggest that there is a need for proper disposal methods for veterinary drug residues and their packaging materials to reduce the potential risks to the environment and wildlife in and around the VNP.

### 3.7 Agricultural pesticides

Our survey for potential toxic hazards for wildlife species around VNP in Rwanda also involved studying the use of different pesticides in the area. We found that the most commonly used pesticide was SAFARIMAX^®^ (Dinotefuran 20%), which was used at a level of 88.4% in the agricultural sector. THIODAN 4EC^®^ (Endosulfan) was also widely used, with a frequency of 90.3%. DUDU^®^ (Abamectin 20g/L + Acetamiprid 3%) was used by 68.8% of farmers, while DITHANE M.45^®^ (Manconazeb/ dithiocarbametes) had the highest frequency of use at 96.3%. MBOLEA YA MAJIMAJI^®^ (Nitrogen, Potassium and Phosphorous) as Fertilizer was used by 54% of farmers, and ROCKET (Profenofos and Pyrethroid Cypermethrin) had a frequency of use of 60%. Finally, MILLMAX GOLD^®^ (Cymoxanil 6% + Propineb 70%) was used at a level of 0.2% (Table 6). These findings suggest that the use of pesticides is prevalent in the area, particularly for crop farming such as Irish potatoes (*Solanum tuberosum*) and tamarillo /tree tomatoes (*Solanum betaceum*) which rely heavily on pesticide applications to increase yield and prevent agricultural losses.

**Table 6:** Itemized insecticides commonly used to increase agricultural production around VNP.

**Pesticides**	**Frequency (** ***n*** **)**		**Percentage (%)**	
	**Yes**	**No**	**Yes**	**No**
SAFARIMAX^®^ (Dinotefuran 20%)	358	47	88.4%	11.6%
THIODAN^®^ (Endosulfan)	365	39	90.3%	9.7%
Dudu^®^ (Abamectin 20g/L + Acetamiprid 3%)	278	121	68.8%	31.2%
DITHANE M-45^®^ (80% Mancozeb)	389	15	96.3%	3.7%
MBOLEA YA MAJIMAJI^®^(Nitrogen, Potassium and Phosphorous)	218	186	54%	46%
ROCKET^®^ (Profenofos and Pyrethroid Cypermethrin)	242	161	60%	40%
MILLMAX GOLD^®^ (Cymoxanil 6%+Propineb 70%)	1	402	0.2%	99.8%

### 3.8 The storing, disposing, and factors influencing application of pesticides

Results showed that most of the farmers surveyed have designated storage areas for pesticides (88.80%). However, the study also found that there is a lack of proper disposal practices for unused pesticide and packaging materials. Most of the farmers reported outdoor burning of pesticide containers and residues. The findings of the study suggest that there are several factors that could contribute to presence of high quantities of pesticides in the area around VNP. The data indicates that the harmfulness threshold and date fixed in advance were the most influential factors, with 98.8 and 98.3% of respondents respectively. In terms of initiating the treatment, the majority of respondents (98.3%) relied on the harmfulness threshold and date fixed in advance, with regional surveillance playing a slightly larger role at 99.0%. Respondents also took into account the climate when applying pesticides, with 1.5% reporting it as a major contributor to the use of pesticides. In cases where the treatment was ineffective, all respondents (100%) reported increasing the concentration or changing the pesticides used. Only a small percentage (0.2%) of respondents reported consulting a specialist in phytosanitary products.

### 3.9 Poisonous plants

Our study has also revealed several herbal, ornamental, and poisonous plants in the area. In total, 47.9% of respondents reported having herbal plants around their homes. Those herbal plants that were commonly used include Nasturtium (*Tropaeolum majus*), Soap aloe (*Aloe maculate*), Coleus plant (*Coleus kilimandscharica)*, Umutagara *(Crassocephalum multicorymbosum)*, bitter leaf (*Vernonia amygdalina)*, ginger bush *(Tetradenia riparia)*, African soapberry *(Phytolacca dodecandra), and* holy basil *(Ocimumtenuiflorum)*. In addition to herbal plants, the study also revealed the most common toxic plants in that area and they include pyrethrum, Coral tree (*Erythrina genus*), angel trumpet (*Brugmansia genus*), Bracken *fern (Pteridium aquilinum*) and Castor oil plant (*Ricinus Communis*). Other toxic plants identified include Arum lily, *Arizona cypress, cacti*, and Mushrooms (Table 7).

**Table 7:** Poisonous plants found in homesteads around VNP.

**Poisonous plant**	**Frequency (** ***n*** **)**		**Percentage (%)**	
	**Yes**	**No**	**Yes**	**No**
Presence of poisonous plants harmful to people and animals	6	397	1.50%	98.5%
Castor oil (*Ricinus Communis*)	103	300	25.6%	74.4%
Coral tree (*Erythrina genus*)	331	72	82.1	17.9%
Golden dewdrop (*Duranta eracta)*	1	402	0.2%	99.8%
Rhus or Wax tree (*Toxicodendron Succedaneum)*	28	375	6.9%	93.1%
White Cedar tree (*Melia Azedarach*)	1	402	0.2%	99.8%
Angel trumpet (*Brugmansia genus*)	305	98	75.7%	24.3%
Arum lily (*Zantedeschia aethiopica*)	125	278	31%	69%
*Belladonna lily* and *amalylis belladonna*	1	402	0.2%	99.8%
Cacti and other succulents (Adromischus *spp*)	63	340	15.6%	84.4%
Dumb cane (*dieffendenbachia genus)*	12	391	3%	97%
*Euphorbia genus*	2	401	0.5%	99.5%
Arizona cypress (*Hesperocyparis arizonica*)	122	281	30.3%	69.7%
Mushrooms and toadstools	110	293	27.3%	72.7%
Bulbs	2	401	0.5%	99.5%
Sticky weed or asthma weed *(Parietaria Judaica)*	1	402	0.2%	99.8%
Bracken fern (*Pteridium aquilinum*)	226	177	56.1%	43.9

### 3.10 Awareness about potential toxic hazards to wildlife species around VNP

The results of our study indicate that the level of awareness about toxic hazards to wildlife around VNP varied among study participants. The majority of the participants had moderate knowledge (57.10%), while 28.8% insufficient knowledge (that chemicals and natural plant toxins can harm wildlife. However, a smaller percentage of participants was aware (14.10%) (Table 8).

**Table 8:** Awareness about toxic hazards to wildlife species around VNP.

**Knowledge level**	**Frequency (*n*)**	**Percentage (%)**
Insufficient	116	28.8%
Moderate	230	57.1%
Sufficient	57	14.1%

### 3.11 The association between education background of respondents and training on awareness of potential toxic hazards to wildlife in the area around VNP

The findings of our study showed a significant association between the educational background of respondents and their knowledge about potential toxic hazards for wildlife species around VNP. It was observed that the majority of respondents who had attended primary school had moderate knowledge (52.6%) about potential toxic hazards, followed by those who had attended secondary school (76%). On the other hand, respondents without formal education had the lowest proportion (20%). Interestingly, respondents who had attended college or university had sufficient knowledge (50%) compared to those who had attended primary or secondary school. The study also found that respondents who had received training on potential toxic hazards had better knowledge compared to those who had not received any training. Specifically, those who had received training had a higher proportion of respondents with moderate knowledge (60.5%) compared to those who had not received training (43.4%) (Table 9).

**Table 9:** Association between education background of respondents, training on toxic hazards and knowledge toward potential toxic hazards for wildlife species in area around VNP.

**Respondent's characteristics**	**Knowledge**			
	**Insufficient**	**Moderate**	**Sufficient**	***P-*** **value**
**Educational background**				< 0.0001
No formal education	30/90 (33.3%)	42/90 (46.7%)	18/90 (20%)	
Primary	73/209 (34.9%)	110/209 (52.7%)	26/209 (12.4)	
Secondary	13/100 (13%)	76/100 (76%)	11/100 (11%)	
College and University	0/4 (0%)	2/4 (50%)	2/4 (50%)	
**Specific training on potential toxic hazards of pesticides**				0.02
No	33/84 (39.3%)	36/84 (42.9%)	15/84 (17.8.7%)	
Yes	83/319 (26%)	193/319 (60.5%)	43/319 (13.5%)	

## 4 Discussions

The VNP is home to endangered wildlife species such as the mountain gorillas. The GoR is planning an expansion of the VNP to increase habitat for this endangered species. Mountain gorillas, like other wildlife, are susceptible to poisoning from natural and man-made chemicals. For example, pesticides, including rodenticides, pose significant risks to wildlife (22, 24, 25). Pharmaceutical products and household chemicals released into the environment may also pose threats to wildlife directly or indirectly (26, 27). Herbal or poisonous ornamental plants can also affect wildlife negatively (28). Because people in the potential VNP expansion zone use various chemicals, fertilizers, pharmaceuticals, ornamental plants, etc.; for their livelihoods, it is important to determine potential toxic hazards to wildlife so that the area can be prepared before wildlife are introduced to the area. Furthermore, although the park expansion has not yet commenced, wildlife species in VNP, including buffaloes and mountain gorillas, continue to venture out of the park and into surrounding agricultural fields. This ongoing interaction between wildlife and human settlements increases the risk of these animals being exposed to toxic hazards within the community, posing potential threats to their health and wellbeing. Therefore, this study tackles that too even before park expansion program.

This study has identified four main categories of potential toxic hazards to wildlife. These include household chemicals, pharmaceutical products, pesticides, and poisonous plants. Interestingly, survey results revealed that households in the vicinity of the VNP had a low level of house chemical usage in their homes. This is likely due to a number of factors, including the remote location of the park, the limited availability of household chemicals and the cultural practices of the local people. This is good news as it reduces the chances for environmental contamination. However, use of dishwashing detergents and laundry detergents was observed at varying levels. Some detergents have extreme pH values and exposure to such detergents is corrosive and can cause caustic injury on contact with mouth or skin (29).

Our study also uncovered evidence of significant use of rodenticides in the area. Those include zinc phosphide and sodium monofluoroacetate. However, zinc phosphide was more prevalent. Zinc phosphide, primarily used as a rodenticide, poses a threat to local wildlife because it releases toxic phosphine gas, impacting not only the targeted rodents but also potentially harming non-target species, including humans. All species of animals are susceptible to zinc phosphide poisoning, but avian species, are the most seriously affected and even resulting in death (30). Meanwhile, sodium monofluoroacetate, known as Compound 1080, a potent pesticide used for pest control, can disrupt cellular metabolism and result in organ failure in various animals, making it a substantial risk to both wildlife and humans alike (31, 32).

Rodenticides are widely employed to control rodent populations and to minimize food damage during storage. Rodenticides can affect primary and secondary targets. Some are specific and others are nonspecific. The potential hazards associated with rodenticides can extend beyond their intended targets. Secondary poisoning resulting from the consumption of poisoned rodents by some wildlife (carnivores) and is a well-documented concern. Poisoning on non-target species such mountain gorillas, through ingestion of rodenticides residues or packaging materials is also a serious concern. Additionally, scavengers and other non-target animals may also be exposed to the lethal effects of rodenticides, leading to ecosystem disruptions and unintended consequences for the entire wildlife community.

The high prevalence of the use of human and veterinary pharmaceutical drugs in the area surrounding VNP is a cause for concern. Poorly disposed of pharmaceuticals may be directly accessed and consumed by wildlife, and can easily enter the ecosystem, primarily through wastewater, and reach various surface water bodies such streams, rivers, lakes, wetlands, reservoirs, creeks, and oceans. These drugs may also contaminate ground water sources (33, 34). Primary medicines and their metabolites may also enter the food chain (35). Ultimately, this could harm wildlife including the endangered mountain gorillas. For example, NSAIDs like Ibuprofen are toxic to a wide variety of species (36, 37). Moreover, excessive and inappropriate use of pharmaceuticals, particularly antibiotics in humans and animals, contributes significantly to global antimicrobial resistance (AMR). This misuse leads to antibiotic-resistant bacteria in both groups, endangering public and animal health. In animal agriculture, resistant bacteria can transfer to humans and wildlife through food or the environment, worsening the AMR issue. One of the medications used for treating parasitic infections in livestock was ivermectin. Accidental ingestion of ivermectin by wildlife, whether through contaminated food sources or environmental exposure, can result in a range of toxic effects, including neurological symptoms like tremors and seizures, gastrointestinal disturbances, respiratory distress, ataxia, muscle weakness or paralysis, and lethargy (38–40).

The high frequency of pharmaceutical drug use in the area of the study suggests that there is a potential for mountain gorillas and other wildlife to be exposed to pharmaceutical drugs through a variety of pathways, such as direct ingestion, indirect ingestion through contaminated food or water, or through contact with contaminated surfaces. The results of the survey on the disposal methods of household chemicals and pharmaceutical drugs in the zone surrounding VNP provide crucial insights into potential toxic hazards that may impact the habitat of the endangered mountain gorillas. Alarmingly, results showed a significant proportion of respondents reported poor disposal of pharmaceuticals. They simply throw them away without an adequate disposal. When VNP is expanded it is recommended that proper procedures for disposal of human and animal pharmaceutical drugs is implemented so that wildlife do not get into contact.

Our survey indicates that the use of agricultural insecticides is widespread, with several products being applied at high frequencies. Most of these are potent acetylcholinesterase inhibiting organophosphorus and carbamate pesticides. They are known to be highly toxic to wildlife (41, 42). Ingestion of improperly disposed of these agricultural insecticides not only can kill the primary victim but also causes relay toxicosis which can devastate ecosystems. For example the intentional baiting of carcasses by poachers has killed mammals and vultures simultaneously (43, 44). Therefore, since the use of agricultural insecticides is prevalent, these chemicals must be collected and properly disposed of by authorities before wildlife is allowed access to the expansion zone.

Natural toxins can poison wildlife. Plant toxins are particularly hazardous to wildlife. Therefore, the discovery of herbal, ornamental, and poisonous plants in the study area raises concerns about the potential risks they pose to the wildlife in VNP, including the mountain gorillas. The prevalence of herbal plants around the homes of local communities is notable, with 47.9% of respondents reporting their presence. While some of these plants are likely beneficial (herbal plant species), toxic plants including the Coral tree, Angel trumpet, Bracken fern, and Castor oil plant (Ricin), and others can be lethal to mammals (45). Pyrethrum, a source of pyrethrin insecticides is commercially grown in the area. Wildlife grazing these plants, which have the potential to grow wild if not controlled, may be hazardous., Ricin (*Ricinus communis*) is a potent toxin which inhibits the synthesis of proteins within cells and can cause severe vomiting, diarrhea, seizures, and death (46). These and other toxic plants identified should be eliminated before this area is accessible to wildlife.

The study also reveals varying levels of awareness by survey participants about natural and chemical hazards to wildlife in areas surrounding VNP. While a significant proportion had moderate knowledge, 28% of the population had insufficient knowledge about the dangers of toxicants they pose to wildlife. This suggests that it is necessary to conduct an education campaign to educate the population about dangers natural and chemical toxicants pose to wildlife. That way the public can assist VNP staff in eliminating toxic hazards identified in this survey before they are relocated. In addition, it is necessary to educate communities surrounding VNP about such dangers to enhance their awareness about these toxic hazards to wildlife. Communities that are aware of toxic hazards can play a proactive role in safeguarding the park's ecosystem and the wellbeing of its precious inhabitants, including the endangered mountain gorillas.

In conclusion, this is the first report of toxic hazards in the zone surrounding the VNP which has been identified for future expansion of the park. This research has revealed the presence of various toxic hazards, including household chemicals, human and veterinary pharmaceutical drugs, agricultural insecticides, and poisonous plants. The VNP is a crucial habitat for the endangered mountain gorillas and other wildlife. More research is recommended to fully document the quantity of toxic hazards identified in this survey. Also, in addition to environmental sampling and laboratory analysis of water bodies, soil, river sediments and poisonous plants, is recommended to fully understand whether use of agricultural chemicals for example have contaminated and negatively impacted the ecosystem. This will require collaborative efforts between health care veterinary professionals, agronomists, local communities, wildlife conservationists, chemists and other professionals. Ultimately, this work will improve our understanding of whether and how these pharmaceuticals, household chemicals, pesticides and poisonous plants impact the delicate ecological balance of VNP. This is a one health issue, crucial for maintaining the health of people, domestic animals and wildlife species such as the endangered mountain gorillas that share this unique and fragile habitat; and this requires a one health approach. It is also important to conduct a targeted education campaign to communities surrounding VNP to increase their awareness of the toxic hazards to wildlife. By increasing their knowledge and awareness we can foster a more sustainable relationship between the communities and the natural environment, contributing to the conservation efforts for the mountain gorillas and their habitat.

## Data availability statement

The raw data supporting the conclusions of this article will be made available by the authors, without undue reservation.

## Ethics statement

This study was approved by the University of Rwanda (The office of the director of research and innovation) and Rwanda Development Board (RDB) in charge of wildlife protection. The interviews were conducted in the local language (Kinyarwanda), and verbal consent was obtained from all participants.

## Author contributions

EK: Funding acquisition, Investigation, Writing—original draft, Writing—review & editing. WR: Conceptualization, Supervision, Validation, Visualization, Writing—review & editing. KN: Conceptualization, Funding acquisition, Supervision, Validation, Visualization, Writing—review & editing. JN: Conceptualization, Validation, Visualization, Writing—review & editing.
