# Supplementary material for: A survey to document toxic hazards in the zone surrounding volcanoes national park, a habitat for mountain gorillas, an endangered wildlife species in Rwanda
Source: Front Vet Sci. 2024 Jan 3;10:1320162. doi: 10.3389/fvets.2023.1320162 (PMC10791935; doi:10.3389/fvets.2023.1320162)
Supplement: Supplementary file 1 [file Data_Sheet_1.docx]

## **QUESTIONAIRE**

**SECTION A: GENERAL QUESTIONS FOR ALL RESPONDENTS**

### **Demographic profile of the respondents**

1. Age (years): _____________

2. what is your occupations _____________

3. Farming experience (year): ______________

4. Sector

1. Kinigi
2. Shingiro
3. Gataraga

5.Family situation

1. Single
2. Married
3. Widower
4. Divorced

6. Family size: (number of persons) …………………….

7. Education level

1. Illiterate
2. Primary
3. College
4. Secondary
5. University

**14 key questions**

The questionnaire consisted of questions but 14 key questions where selected to understand the level of Knowledge of respondents towards potential toxic hazards for wildlife species in area around Volcanoes National Park. The level of knowledge of the respondent was analyzed based on their knowledge on responses provided on key questions such as *(i) Do you know that the household chemicals are harmful to humans and environment as well? (ii) Have you ever experienced in your family any of the following symptoms applying household chemicals? (iii) Do you know that environmental pollution is linked to the application of household chemicals? (iv) Do you know the relationship between of household chemicals and disease? (v) Do you know and have any plant at or around your home among the following? (vi) Do you know the pesticide residues? (vii) Do you know that the pesticides are harmful to humans? (viii) Have you ever experienced in your family any of the following symptoms applying pesticides? (ix) Do you know that environmental pollution is linked to the application of pesticides? (x) Do you know the relationship between pesticides and disease? (xi) Do you know pharmaceutical products residues? (xii) What is the fate of the pharmaceutical product residues after its use? (xiii) What is the fate of the sprayer rinse water after its use? (xiv) What is the fate of chemical packaging?*

### **Household chemicals**

**What are the household chemicals that are used by people in the area that surround VNP?**

1. Do you use chemicals in bathroom and toilet at your home?

Yes

No

2. If so, choose any of the following (Select all that apply)

1. Medicines (if so what kind?)
2. Disinfectants and surface cleaners
3. Deodorant blocks and sprays
4. bleach
5. perfumes
6. nail polish and nail polish remover
7. hair dyeing and hair styling products
8. hair removers.
9. Other (Please specify)

3. May you please list those medicine?

1. .
2. .
3. .
4. .
5. .
6. .
7. .
8. .

4. Do you use chemicals in kitchen at your home

1. Yes
2. No

5. If so, choose any of the following (Select all that apply)

1. dishwashing detergents and powders
2. surface cleaners, sprays and degreasers
3. insect sprays and baits
4. drain cleaners
5. lamp oils
6. medicines (for yourself or your pets)
7. Other (Please specify)

6. Do you use chemicals in laundry at your home

1. Yes
2. No

7. If so, choose any of the following (Select all that apply)

1. clothes washing detergents and fabric softeners
2. bleach
3. solvents
4. drain cleaners
5. pet flea powder and shampoo
6. metal and wood polish
7. washing soda and borax
8. antiseptics
9. Other (Please specify)

8. Do you use chemicals in Garage, storage shed and pool areas at your home

1. Yes
2. No

9. If so, choose any of the following (Select all that apply)

1. petrol
2. methylated spirits, turpentine and white spirits
3. kerosene and lamp oils
4. paint and paint strippers
5. degreasers
6. rust removers
7. acids
8. lubricating oils
9. pool chemicals
10. pesticides
11. lime
12. fertilizers
13. Other (Please specify)

10. Do you use chemicals in laundry at your home

1. Yes
2. No

11. If so, choose any of the following (Select all that apply)

1. medicines, such as sleeping tablets, painkillers and cough and cold medicines
2. cosmetics and perfumes
3. insect repellents
4. Other (Please specify)

12. Do you apply any of the following chemical in the house or around the house at your home (Select all apply)

1. Insecticides
2. Rodenticides
3. Herbicides
4. Pesticides
5. Other (Please specify)

13. How do you dispose those chemical residues or chemical package materials after use?

1. Throw it away
2. Wrap in separate container before throwing away
3. Burial
4. Other (Please specify)

14. How often do you use any of the chemicals at your home?

1. More than once per week
2. Once per week
3. Once per month
4. Once every 3 months
5. Once every 6 months
6. Once per year

**What is the household member’s awareness of the Dangers of household chemicals to Human Health and Environment?**

1. Do you know that the household chemicals are harmful to humans and environment as well?

1. Yes
2. No

2. If so, have you ever experienced in your family any of the following symptoms applying household chemicals (Select all that apply)

1. tearing eyes
2. burning of the eyes, nose, throat, chest and skin
3. headache,
4. sweating,
5. blurred vision,
6. stomach aches and
7. diarrhea

3. Do you know that environmental pollution is linked to the application of household chemicals?

1. Yes
2. No

4. Do you know the relationship between of household chemicals and disease?

1. Yes
2. No

### **POISONAOUS PLANTS: HERBAL AND OR ARNAMENTAL PLANTS**

**What are plants that may be poisonous which are found at and or around home in the area that surround VNP**

1. Do you have herbal plant at your home

1. Yes
2. No

2. If yes, may you please list all of them

1. .
2. .
3. .
4. .
5. .
6. .

3. Do know and have plants that are harmful to human and animal when they are consumed.

- Yes
- No

4. If so, may you please list all of them

1. .
2. .
3. .
4. .
5. .

5. Do you know and have any plant at or around your home among the following (Select all that apply)

1. Castor oil plant (*Ricinus communis*) (Ikibononono)
2. Coral tree (*Erythrina genus*)
3. Common or pink aleander and yellow oleander
4. Deadly nightshade (*Atropa belladonna*)
5. Golden dewdrop (*Duranta erecta*)
6. Rhus or wax tree (*Toxicodendron succedaneum*)
7. White cedar tree (*Melia azedarach*)
8. other

6. Do you have ornamental plants in or around houses

1. Yes
2. No

7. If so, may you please list all of them

1. .
2. .
3. .
4. .
5. .
6. .

**SECTION B: IF A RESPONDENT IS A FARMER**

1. What is the type of farming do you do

1. Crop farming
2. Livestock farming

**B. 1. If you practice crop farming.**

1. Size of farm land (hectare) _____________

2. Farmer status

1. Large farmer
2. Medium farmer
3. Small farmer

**What are the commonly Used agricultural chemicals that are applied by farmers in the area that surround VNP?**

1. Do you use pesticides to combat diseases and increase production?

1. Never
2. Rare
3. Often

2. If so, please list ALL pesticides used over the last 12 months?

1. ……..
2. ……..
3. ……..
4. ………

3. Describe how do you store pesticides used in agriculture

4. Have you ever received a training on pesticide management, especially using personal protective equipment during application?

1. Yes
2. No

5. If yes, who provided the training?

1. RAB Extension workers
2. Others (Please specify)

6. How do you dispose the expired pesticide?

1. Application in the treated field.
2. Application on uncultivated land
3. Release into waterways

**What is the knowledge and decision-making mechanism related to pesticide use practices?**

1. To initiate treatment, you rely on?

1. Harmfulness threshold
2. The date fixed in advance
3. Regional surveillance
4. Neighbors
5. Other (please specify)

2. What support is available for decision making in terms of choice of the **date** of treatment?

1. Harmfulness threshold
2. The date fixed in advance
3. Regional surveillance
4. Neighbors
5. Other (please specify)

3. What support is available for decision making in terms of choice of the concentration of treatment

1. Harmfulness threshold
2. The date fixed in advance
3. Regional surveillance
4. Neighbors
5. Other (please specify)

4. What support is available for decision making in terms of choice of the pesticide**?**

1. Harmfulness threshold
2. The date fixed in advance
3. Regional surveillance
4. Neighbors

5. If treatment is ineffective, do you?

1. increase the concentration or change the pesticide.
2. consult a specialist in phytosanitary products

6. Where do you buy phytosanitary products?

1. Retailer
2. Company directly

7. Do you respect the recommended use of concentrations?

1. Often
2. Frequent
3. Always

8. How do you measure the right dose for application?

1. Follow recommended dose
2. Do not follow recommended dose

8. Do you take into account the climate (rain, wind ...) to carry out a treatment?

1. Yes
2. No

9. Do you take into account the time of day (morning, midday, evening) when performing treatment?

1. Yes
2. No

10. Who’s in charge of treatment?

1. Myself
2. Qualified person
3. Simple farmer worker

**What is the Farmers’ Awareness of the Dangers of Pesticides to Human Health and Environment?**

1. Do you know the pesticide residues

1. Yes
2. No

2. Do you know that the pesticides are harmful to humans

1. Yes
2. No

3. If so, have you ever experienced in your family any of the following symptoms applying pesticides (Select all that apply)

1. Nausea/Vomiting
2. Visual disturbances
3. Dizziness
4. Headache
5. Excessive sweating
6. Respiratory problems
7. Excessive salivation
8. Convulsion

4. Do you know that environmental pollution is linked to the application of pesticides?

1. Yes
2. No

5. Do you know the relationship between pesticides and disease?

1. Yes
2. No

**What is the Behavior of farmers for storing and disposing of pesticides?**

1. Do you have a room fitted out for the storage of phytosanitary products?

1. Yes
2. No

2. What is the fate of the sprayer rinse water after its use?

1. Application in the treated field.
2. Application on uncultivated land
3. Release into waterways

3. What is the fate of chemical packaging?

- Leave at the edge of the fields

1. Yes
2. No

- Burial

1. Yes
2. No

- Public landfills

1. Yes
2. No

- Streams

1. Yes
2. No

- Cremation

1. Yes
2. No

**What are Precautionary measures used by the farmers while and after applying pesticides.**

1. How often do you use waterproof gloves

1. Never
2. Sometimes
3. Every use

2. How often do you use of hat

1. Never
2. Sometimes
3. Every use

3. How often do you use masks

1. Never Sometimes
2. Every use

4. How often do you use boots

1. Never
2. Sometimes
3. Every use

5. how often do you use mask with filter cartridge

1. Never
2. Sometimes
3. Every use

6. How often do you use goggles

1. Never
2. Sometimes
3. Every use

7. Do you Consume food while spraying

1. Yes
2. No

8. Do you Consume drinks while spraying

1. Yes
2. No

9. What types of measures do you take after application pesticides?

- Take a shower

1. Yes
2. No

- Cleaning clothes

1. Yes
2. No

**B.2 If you practice livestock farming**

1. what is your farm status

1. Small
2. Medium
3. Large

2. how often your livestock get sick

1. Very often
2. Rare
3. Never

3. If so, what type of treatment do you use

1. Traditional
2. Pharmaceutical products

4. If so, please list all pharmaceuticals that you have applied in previous last 12 months

1. .
2. .
3. .
4. .
5. .
6. .
7. .
8. .
9. .

5. Do you apply acaricides on your livestock

1. Yes
2. No

6. If so, may you list the types of acaricides that you use please?

1. .
2. .
3. .
4. .
5. .
6. .

**What is the Behavior of farmers for storing and disposing of pharmaceuticals.**

1. Do you have a room fitted out for the storage of phytosanitary products?

1. Yes
2. No

2. Do you know pharmaceutical products residues

1. Yes
2. No

3. What is the fate of the pharmaceutical product residues after its use?

1. Application in the treated field.
2. Application on uncultivated land
3. Release into waterways

4. What is the fate of pharmaceutical product packaging?

- Leave at the edge of the fields

1. Yes
2. No

- Burial

1. Yes
2. No

- Public landfills

1. Yes
2. No

- Streams

1. Yes
2. No

- Cremation

1. Yes
2. No
